# Lectures on the Treatment of Wounds

**Published:** 1843-01-21

**Authors:** 

**Affiliations:** Paris


THE MEDICAL EXAMINER,

And Retrospect of the Medical Sciences.

Vol. VI.] PHILADELPHIA, SATURDAY, JANUARY 21, 1843. [No. 1.

### LECTURES ON THE TREATMENT OF WOUNDS.

*Delivered at the Hôtel Dieu, of Paris.*

BY M. ROUX.

### LECTURE I.

[Translated for the Medical Examiner.]

Gentlemen: Wounds constitute nearly one-half of the cases which occur in our surgical wards, and which hereafter you will be called on to treat, either in hospital or private practice. A proper knowledge of their treatment becomes, therefore, an object of primary importance. I shall not here occupy your time in general remarks, with which you are already familiar, or which you will find in all the didactic treatises; but I shall confine myself to the consideration of practical points of great utility, and but too much neglected. I shall first speak of simple or uncomplicated wounds, reserving for a future occasion the consideration of the other classes of wounds. Simple wounds may be divided into five varieties, or classes:

1. Accidental wounds, properly so called.
2. Wounds following operations.
3. Wounds following abscesses.
4. Wounds resulting from sloughing of the soft parts, or from the elimination of sequestra, which could not appear externally without causing a solution of continuity more or less regular, of which nature attempts the restoration.
5. Lastly, all ulcers, or fistulous openings, which being maintained by some special cause, have, on its removal, been converted into wounds, and have a tendency to cicatrise.

There are also a class of wounds which undergo successive transformations; which, from having been simple wounds, take on the character, from bad treatment, or some especial cause, of ulcers and fistulæ; and, from the intervention of art, resume their original character. Such are the different varieties of wounds, in which nature herself undertakes the cure, and where we should only interfere to second her intentions.

The two first species may be regarded as primitive. no pathological state having preceded them; whereas, all the rest are rather secondary, it being necessary to remove some morbid condition before we can reduce them to a simple state. Between these two orders of wounds there is a striking and important difference, to which I must, for a moment, call your attention.

The distinctive character of the first, or primitive class of simple wounds, is there being, in general, regular with hæmorrhage, and requiring a certain lapse of time for the development of sufficient adhesive inflammation to produce cicatrization. The second class of simple wounds bleed usually but little, suppurate, and take on adhesive inflammation but slowly, from their chronic tendency. They only lutimately heal by a process entirely different from that which effects union in the preceding class.—This difference in their nature produces also an essential difference in their mode of treatment, upon which I shall particularly dwell hereafter. This difference is especially well marked at their commencement. Primitive wounds differ also among themselves, depending on their cause, whether an accident or operation. Wounds following operations are in general more profound and extensive, and embrace a greater loss of substance, than those which are occasioned by extreme violence, if we except some cases of gunshot and lacerated wounds.—Besides, wounds which are made artificially, are neater, more regular, in a word, more graceful, (*plus gracieux,*) if I may be permitted the expression; they are always simple, without contusion or laceration; whereas, most accidental wounds are more or less irregular and bruised. Wounds, too, made by surgical instruments, are commonly not complicated by the presence of foreign substances, if we except those cases where we are forced, temporarily, to introduce, therapeutically, certain foreign bodies, as ligatures, for example, in the midst of divided tissues. Nor are they influenced by special causes, which may retard cicatrization. Wounds resulting from operations are always more favourably disposed to cicatrize than any others, because we can treat them without reference to particular causes, which oblige us to adopt a special method, which often happens in accidental wounds. Hence my astonishment, that the practice of healing wounds speedily by immediate union has been so reluctantly adopted by French surgeons, and that they have for such a length of time shown such hostility to it. For ages, surgeons amused themselves by permitting simple wounds to suppurate for an indefinite period; and there are, even at this present day, practitioners who obstinately pursue the old routine, and deny all the advantages of immediate reunion.

Let us now say a few words upon the other varieties of wounds which we have called secondary. Wounds of the third order, which succeed to abscesses, in spite of the efforts of nature towards cicatrization, encounter great obstacles towards healing, in the thinning of the integuments, and in the destruction of the subcutaneous cellular tissues, all counter-indications to the rapid progress of cicatrization. Everybody knows that the more superficial an abscess is, or the later it is in opening, the more difficult it becomes to obtain a complete cure, for causes which I will mention presently. To overcome these inconveniences, I have adopted a particular mode of opening abscesses, which is pursued by but few practitioners. I incise largely, without sparing the adjoining parts; I make my incisions sometimes in T, or in X, and that for a regard of the future, being convinced that, by this method, the portions of attenuated skin, which left in contact would never unite, retract on being largely opened, and cicatrization takes place from the bottom of the wound, and becomes completed in far less time than if the incisions had been small. When, on the contrary, you wish to spare your patients these large and deep incisions, you run the risk of exposing them to consecutive operations, more painful and often more dangerous.

Wounds of the fourth class, following gangrene, are distinguished from others by being ordinarily irregular, with more or less loss of substance, the inevitable result of the separation of the eschars. These wounds have such distinctive peculiar characters, that it is only sufficient to see them, after a little practice, to recognize them. As to those wounds which are the consequence of the action of external agents, such as fire, acids, &c., their physiognomy generally depends on the nature of the substances which produced them. Thus, those which follow burns have a strong tendency to the production of vegetations, and their cicatrices are in general irregular, and have an aspect altogether characteristic. The same may be said of those caused by caustics. Thirty years ago, the ordinary caustic employed was the arsenical paste; the eschars resulting from the application of this caustic are tenacious, slow in separating, and the cicatrix is formed beneath it, so that on its separation, you find the wound nearly entirely healed, handsome and regular. It is for this reason that modern surgeons, who employ caustics in the treatment of cancerous affections, prefer the arsenical paste. These remarks apply also to other wounds; to those, for example, which have a soft and fungoid appearance. Within the past few days, I have called your attention in the wards to the peculiar appearance of the wound of a breast which I amputated for a cancer some time ago. In this woman the skin seems to have an especial disposition to melanosis. In spite of this, the wound advanced rapidly towards union, against all my endeavours, and never presented a healthy suppuration; the pus, on the contrary, was badly constituted, and the wound, always bloody, had a fungous aspect, resembling that following the ablation of sanguineous tumours, appearances which made me fear a delay in the process of cicatrization.

I have frequently removed large sanguineous tumours, always being careful to carry my incisions well into the sound integuments, and I have obtained wounds of a good appearance, but I have never been able to obtain a healthy cicatrization; they always becoming, after some time, soft and bleeding on the slightest touch. The cicatrix is not less firm, but presents a peculiar character. I have seen this so frequently, that now I have acquired the habit of distinguishing them at first sight, by the appearance and form of the cicatrix. I was once able to remark this peculiarity five times in the same individual—a young person—who had five fungous tumours. I removed them at an interval of some days, and I found in all the resulting wounds the same character, the same progress, and the same mode of cicatrizing. I saw him some years subsequently, and the cicatrices, which remained perfectly sound, presented the same peculiar aspect.

Let us now consider the treatment of the two classes of wounds, primitive and secondary, of which we have been speaking. The first variety allows of two different methods of treatment, according to the condition: whether it is recent, with a tendency to immediate adhesion, or whether suppuration is established. In the first case, we can obtain union by the first intention; in the second, mediate union can only be obtained, by the suppurative process at a period more or less remote. When suppuration is very recent, and there exists sufficient inflammation to produce adhesion of the edges of the wound, we may ultimately have immediate union. It is from this fact that an Irish surgeon has derived his *secondary reunion;* that is, always waiting for suppuration to commence before attempting union. That this practice, under certain circumstances, presents advantages, cannot be denied.

Primitive wounds admit, then, of two methods of treatment: let us examine them.

Union by the first intention offers, certainly, incontestible advantages; but is not entirely exempt from inconveniences. It is necessary to know when to resort to it. An ardent desire to study and comprehend English surgery, led me to London in 1814. Our neighbours have certainly given us many good therapeutic ideas, and we have profited by them, but their practice is not always above reproach. One day I assisted Sir Astley Cooper, then at the height of his fame, in an operation for sarcocele, and saw him bring the edges of the wound together by sutures. This method of dressing struck me as very improper, because the tissues were thin and soft, and could hardly be maintained in apposition long enough for cicatrization to proceed regularly. The consequences I foresaw happened in a few days—gangrene supervened, and with great loss of substance. We should, therefore, exercise a just discretion in the employment of this method, and not use it indiscriminately. It is, without doubt, a very fortunate thing to obtain, in a short time, the cicatrization of a wound, and in this point of view, no one will contest the advantages of immediate reunion, and it is also to this method that surgery owes some of its most brilliant successes. It is now known that in the earliest ages this method was practised, and it is really surprising that its advantages were so long neglected. The time, too, is not very long when the first surgeons amongst us opposed this practice with all their might. It is about twenty-five years since this practice was revived, and even now many surgeons vehemently oppose it. For my part, I have contributed much towards its adoption and popularization in France, at a time when every one opposed it, but when it was in England in all its force.

What has been said of immediate union applies also to the suture, which constitutes one of the steps of this method, and one of the most efficacious of our means of union. The suture is also a very old mode of union, and one which has never been justly appreciated. In France, from the time of the Academy of Surgery, it was but little employed, its dangers being much exaggerated. Louis, who lent such a renown to surgery at this time, opposed it violently in every variety of wounds, even after the operation for hare-lip. And yet how is it possible to obtain, after this operation, a neat and regular cicatrix without some points of suture, which will keep the edges in close apposition? In spite of the talent and influence of the opposition, time has done justice to these unfortunate prejudices, and experience has fully shown that this practice in nowise entails the inconveniences which Louis attributed to it, whenever it is employed with the necessary precautions. If the inconveniences and dangers were so great as to force us to give them up, we should be obliged to abandon a crowd of important and beautiful operations. What, without the suture, for example, would become of plastic surgery? I have re-made, on the same individual, nearly an entire cheek, an upper lip, and nose, by borrowing from the neighbouring parts a sufficient extent of integument. How could I, without sutures, have maintained in place these large flaps of cutaneous tissue? Let us acknowledge, then, the real and great advantages of this means of union; but, at the same time, let us be on our guard against the abuses which some surgeons indulge in, and employ it ourselves with discernment.

*Paris*, November, 1842.

